# Stickleback mass occurrence driven by spatially uneven parasite pressure? Insights into infection dynamics, host mortality, and epizootic variability

**DOI:** 10.1007/s00436-022-07517-4

**Published:** 2022-04-18

**Authors:** Jan Baer, Sarah M. Gugele, Samuel Roch, Alexander Brinker

**Affiliations:** 1 Fisheries Research Station Baden-Württemberg, Argenweg 50/1, 88085 Langenargen, Germany; 2 Institute for Limnology, University of Constance, Mainaustraße 252, 78464 Konstanz, Germany; 3 Division Agriculture and Rural Area, Department Fisheries and Water Ecology, State Government of Vorarlberg, Auhafendamm 1, 6972 Hard, Austria

**Keywords:** *Gasterosteus aculeatus*, Infection, Invasion, Life history, Parasitism, *Schistocephalus solidus*

## Abstract

Since 2012, a massive invasion of the three-spined stickleback (*Gasterosteus aculeatus*) has taken place into the pelagic area of Lake Constance. This species, which had previously been restricted to the littoral zone, is now the dominant pelagic fish and the previously dominant whitefish (*Coregonus wartmanni*) has suffered severe reductions in growth and recruitment. In this study, in total, 2871 sticklebacks were collected via monthly sessions over a 4-year period in pelagic and benthic areas of Lake Constance and examined for signs of infection with *Schistocephalus solidus*, a parasite known to be potentially fatal. The infection risk to sticklebacks increases throughout the course of the year and is size- and sex-dependent. Habitat has only a marginal impact. All parasite-induced harm is imparted after stickleback spawning and parental care is over. The results did not support the hypothesis that the invasion of the pelagic area might be driven by parasite-avoiding behaviour. Furthermore, the impact of the parasite is likely to be limited to post-reproductive adults, thereby ensuring stable reproduction of the hosts despite high rates of transmission and mortality. In consequence, stickleback stock development is independent of *S. solidus* infection, leading to secure coexistence of host and parasite even at extraordinary high host levels.

## Introduction

Invasive species can have detrimental effects on natural habitats and in extreme cases an invasion can impact entire ecosystems and food webs (Didham et al. 2005). One such case is the invasion of one of the largest lakes in Central Europe, Lake Constance, by the three-spined stickleback (*Gasterosteus aculeatus* L.) (hereafter referred to as stickleback). The species first was established in the littoral zone of Upper Lake Constance (ULC) around 1958 (Muckle 1972; Roch et al. 2018). However at the end of 2012, it expanded from an exclusively shoreline habitat into the pelagic zone (Eckmann and Engesser 2019). The reason for this behaviour is unclear and no evidence exists that before 2012 sticklebacks from other origin (e.g. sticklebacks from the Baltic Sea) were introduced in the lake. Nevertheless, a lake-wide fishing survey of ULC in September 2014 revealed that sticklebacks represented 96% of all fish in the pelagic fish community and accounted for 28% of total fish biomass (Alexander et al. 2016) which was confirmed in 2019 (www.ibkf.org). Furthermore, hydroacoustic surveys of the pelagic zone, conducted twice a year from 2009 to 2018, revealed that the population density of sticklebacks increased exponentially in 2012 up to a plateau with seasonal variations after 2014, with a maximum record of 7990 individuals/ha (Eckmann and Engesser 2019). The full ecological impact of sticklebacks on the aquatic community in ULC and its tributaries is unknown. However, the results of two recent studies (Roch et al. 2018; Rösch et al. 2018) imply significant effects of stickleback presence on pelagic whitefish *Coregonus wartmanni* (Bloch 1784), the previously dominant pelagic fish species and the main target of local fisheries (Baer et al. 2017). The suspected reasons include interspecific competition for food leading to reduced growth and survival, and predation by sticklebacks on whitefish larvae and probably eggs, hampering recruitment (Ros et al. 2019; Baer et al. 2021). These observations coincide with a sharp decline in whitefish yield, from around 300–600 mt (metric tons) before stickleback invasion to less than 150 mt with stickleback presence in the pelagic zone (Roch et al. 2018). In 2019, the yield fell further, to below 60 mt (Gugele et al. 2020). Another theoretical explanation for these decreases could be the invasion of the quagga mussel in 2016 (Werner et al. 2016); however, the impact of the quagga mussel is unclear and occurred some years after the whitefish stock decreased. Additionally, the steep increase of the cormorant population recorded during the last 20 years in this area might have influenced the reduction (www.ibkf.org); however, cormorants forage mainly on other, more littoral living fish species like percids and cyprinids and only rarely on sticklebacks (Suter 1997). Therefore, the increase of stickleback density and their supposed negative impacts (competition and predation of whitefish eggs and larvae) is the most probable explanation for the decreasing whitefish stocks — and the increasing density of cormorants will not lead to a stickleback stock reduction.

Two years after the spreading of sticklebacks into the pelagic zone, the parasite burden of sticklebacks was investigated and already revealed high rates of infection by the pseudophyllidean cestode *Schistocephalus solidus* Müller 1776. Infection brings known consequences for host survivorship, reproductive success, and the possibility of high parasite-induced mortality. Several studies have shown that reduced stickleback survival and reproduction can lead to large fluctuations in population size (Heins et al. 2010), and infection by *Schistocephalus solidus* has previously been linked to significant mortalities of stickleback stock within short periods of time (Pennycuick 1971).

**Fig. 1:**
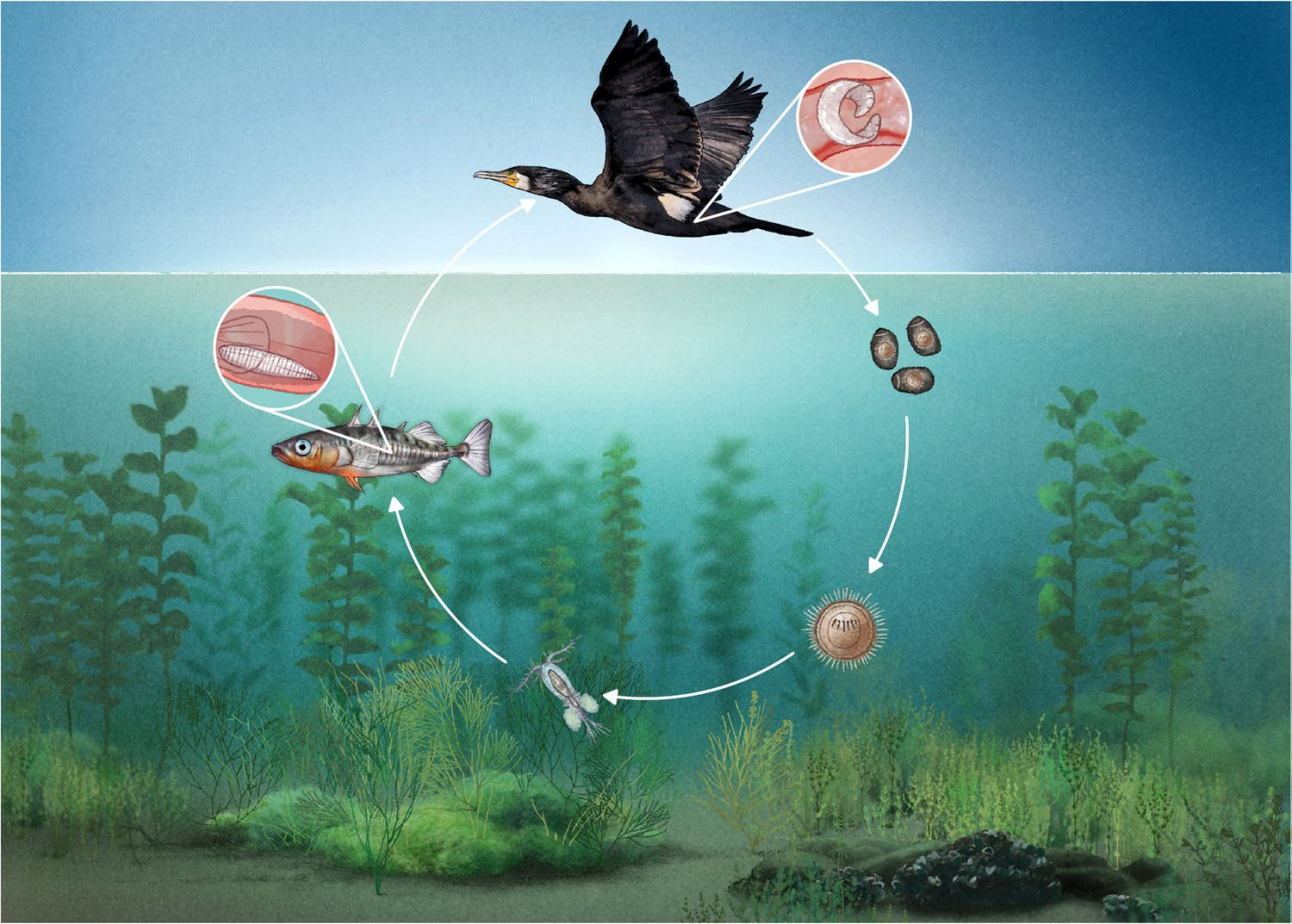
The complex life cycle of *Schistocephalus* *solidus* — explanation see text

The complex life cycle of the tapeworm (Fig. 1) starts with the hatching of a free-swimming larva called a coracidium, which is ingested by a copepod inside which it develops into the next larval stage, the procercoid. In ULC, different taxa of cyclopoid copepods species (*Cyclops abyssorum*,* Mesocyclops leuckartii*, and *Cyclops vicinus*) exist (Straile 2015) and are potential first intermediate hosts of *Schistocephalus solidus* (Wedekind and Milinski 1996) though parasitological investigations are missing. The following larval stage, the plerocercoid, develops after the copepod is ingested by a stickleback, where it parasitises in the visceral cavity. The parasite reaches its adult stage in the intestine of the final host, a piscivorous bird. Notably nearly all the parasite’s growth takes place during the plerocercoid stage, in which mass may come to exceed that of its stickleback host (Pennycuick 1971). Hence, a parasitised stickleback with a full-grown parasite can be recognised macroscopically by a distended abdomen (Schultz et al. 2006). Several studies document direct effects of the parasite on sticklebacks, including changed behaviour or colouration (Tierney et al. 1993; LoBue and Bell 1993; Ness and Foster 1999; Jolles et al. 2020), shifts in morphology (Barber and Svensson 2003), reduced energy stores and somatic condition (Tierney et al. 1996; Barber and Svensson 2003; Bagamian et al. 2004), increased or decreased growth rate (Arnott et al. 2000; Barber and Svensson 2003), reduced fertility (McPhail and Peacock 1983; Tierney et al. 1996; Heins et al. 1999), and decreased egg (Heins and Baker 2003) and gonad size (Tierney et al. 1996).

Today in ULC, the stickleback population occupies two distinct habitats. While some remain in the historical littoral area the whole year, since 2012 a significant proportion of the population inhabits the pelagic waters of ULC during most of the year and only migrate to the littoral habitat for spawning (Gugele et al. 2020). One possible explanation for this unexpected behaviour might be an active avoidance of areas (littoral zones) with high risk of *Schistocephalus* infection via parasitised copepods (MacColl 2009). This hypothesis is supported by laboratory observations that sticklebacks choose actively other water columns in the presence of parasites (Poulin and FitzGerald 2011). Therefore, habitat selection may serve to reduce the risk of parasitism. However, quantitative description of the parasite population in the host (e.g. intensity of infection, parasite size) in dependence on habitat (pelagic vs. littoral zone) is missing. To test the hypothesis that the spreading into the pelagic zone could be induced to decrease the risk of infection with *S. solidus*, the present study examined the infection of benthic and pelagic sticklebacks with *S. solidus* in Lake Constance. The goal was to test whether habitat choice had an effect on *S. solidus* exposure to sticklebacks and therefore on the fitness and survival of sticklebacks. To this end, the percentage of infected fish (prevalence), the mean intensity of infection, and the parasite:host biomass ratio were used to evaluate the possibility of a potential breakdown of the recent massive stickleback population in the future, as has been seen elsewhere (Pennycuick 1971; Heins et al. 2010). Therefore, during four consecutive years, a monthly sampling was conducted to get insight into the autecology of the *S. solidus*–stickleback relationship in one of the largest lakes in Central Europe.

## Methods

### Study area

**Fig. 2:**
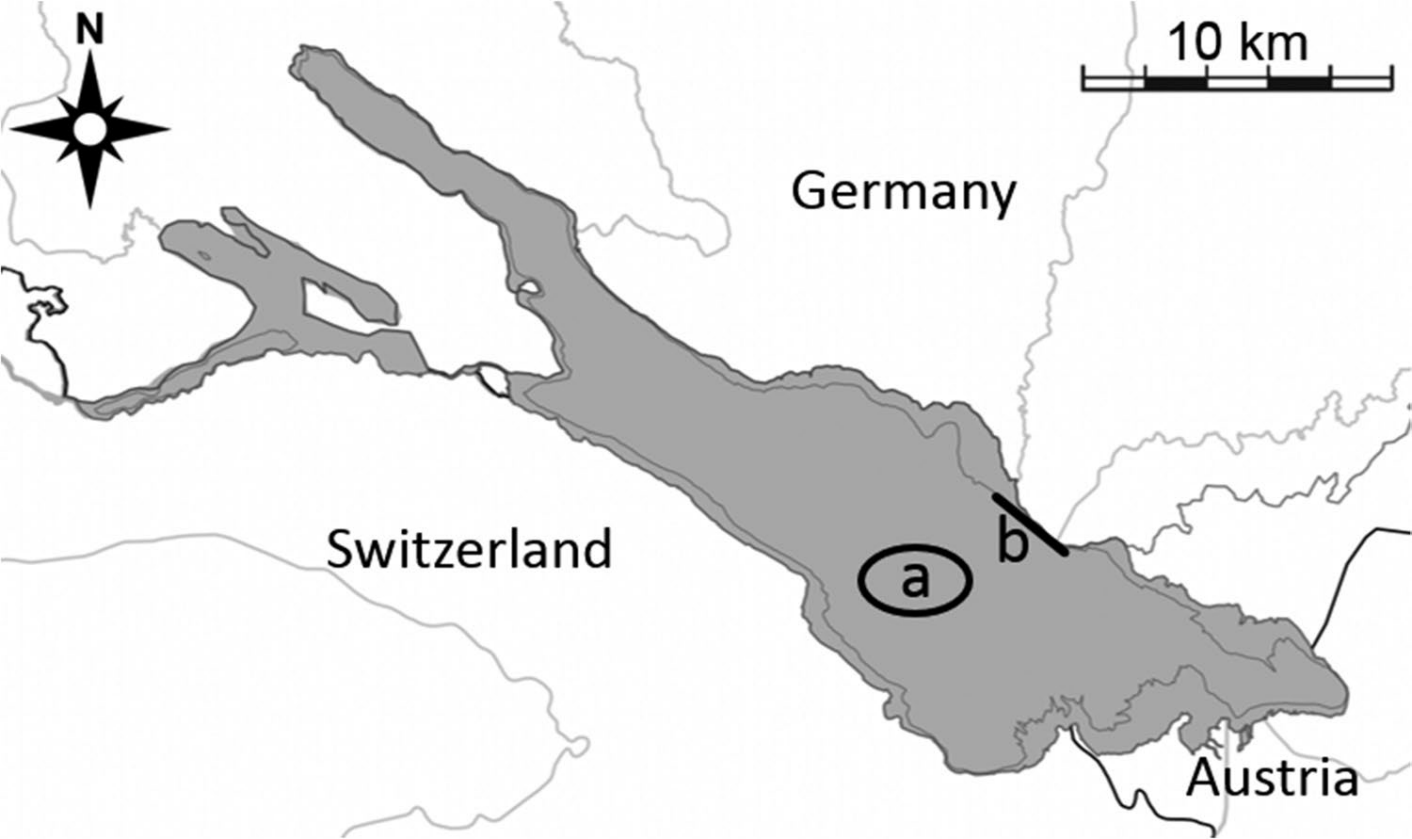
Map showing a) pelagic and b) benthic gillnet sampling locations in Upper Lake Constance

Lake Constance is situated between Austria, Germany, and Switzerland (Fig. 2) and is part of the Rhine drainage basin. It has a surface area of 536 km^2^, of which 472 km^2^ belong to the deep Upper Lake (ULC) and 63 km^2^ to the shallower Lower Lake (LLC). ULC has undergone intensive re-oligotrophication in recent years (Stich and Brinker 2010). The fish community comprises a minimum of 30 species (Eckmann and Rösch 1998), of which about 10 are targeted by its inland fishery (Rösch 2014). Of these, whitefish (*Coregonus* spp.) are the economically most important species, and fisheries management is based on routine monitoring of this important group (www.ibkf.org). An overview of the fisheries situation is given by Baer et al. (2017). This study focused solely on ULC; therefore, only data related to ULC are presented.

### Ethics approval and consent to participate

Approval for the present study by a review board institution or ethics committee was not necessary because all fish were caught by permission of the local fisheries administration (Fisheries administration RP Tübingen). The qualifications of participating personnel (fishing licenses) were checked regularly by the local fisheries administration, too. All sticklebacks were euthanised with an overdose of clove oil (1 mL L^−1^) according to the German Animal Welfare Act (TierSchG).

### Sampling

Sampling was done monthly for 4 years, from March 2016 until September 2019 using gillnets in the pelagic and littoral zone of ULC (Fig. 2). In total, 40 surveys were conducted, and due to internal management decisions February was not included in sampling.

Nets for sampling the pelagic area were placed in the middle of the lake, while nets sampling the benthic area of the littoral zone (hereafter referred to as benthic habitat) were set at the eastern shore of the lake (Fig. 2). Four nets with mesh sizes of 6, 8, 10, and 12 mm were set in the pelagic area whereas in the benthic area only three nets were used, with mesh sizes of 6, 8, and 12 mm. The 10-mm mesh net was omitted to avoid the high bycatch of ruffe (*Gymnocephalus cernua* L. 1758) recorded in preliminary studies. All nets had a height of 3 m, while length varied with mesh size: 30 m for nets with 8 and 10 mm mesh, 10 m for the 6-mm mesh net, and 15 m for the 12-mm mesh net. All pelagic nets were deployed to drift freely behind the nets used in the monthly monitoring of whitefish (mesh sizes 36–44 mm), at depths of 3–15 m according to the areas of greatest stickleback abundance recorded during hydroacoustic surveys (Gugele et al. 2020). Benthic nets were set at depths from 6 to 20 m. All nets were set overnight, with a soak time of about 15 h. Since nets with a mesh size of 8 mm were most efficient in trapping sticklebacks, the catches of these nets were used for calculating the catch per unit effort (NPUE, defined as caught stickleback individuals per m^2^ net).

Haphazard samples of sticklebacks from all nets from both habitats were used for assessing *S. solidus* prevalence each month. Usually 70 sticklebacks per habitat per month were frozen at minus 18 °C and inspected visually for infection later in the laboratory; if fewer than 70 sticklebacks were caught, all individuals were checked. In total, 2872 sticklebacks were analysed (2016: *n* = 673; 2017: *n* = 819; 2018: *n* = 860; 2019: *n* = 519). All fish were measured (total length (*L*) to the nearest mm) and weighed to the nearest 0.01 g and sex was recorded; parasites were counted per host and weighed *en masse* to the nearest 0.01 g after they were blotted. Two thousand sixty-nine of the 2871 analysed sticklebacks were caught in the benthic habitat, and 803 in the pelagic zone. Two thousand one hundred twenty-nine of all analysed fish were females, with a mean *L* of 69 mm (± 7 mm standard deviation s.d.) and a mean wet weight of 3.1 g (± 1.1 g s.d.). Seven hundred four were males with a mean *L* of 65 mm (± 6 mm s.d.) and a mean wet weight of 2.5 g (± 0.8 g s.d.). A further 39 individuals were juveniles with a mean *L* 51 mm (± 4 mm s.d.) and a mean wet weight of 1.3 g (± 0.3 g s.d.).

### Data treatment

Parasitology terms are used according to Bush et al. (1997). Each year was divided into four seasons according to the local climate zone and lake stratification (spring: April to May, summer: June to September, autumn: October to December, winter: January and March) (Stich and Brinker 2010).

Prevalence was calculated as the number of sticklebacks infected with one or more *S. solidus* divided by the number of examined sticklebacks. To test the potential effects of habitat, season, year, *L*, and sex including interactions of season and sex with *L* on prevalence, a nominal logistic regression was used, using the status of infected fish as nominal data (infected yes or no). To assess main effect importance, the effect strengths were calculated using Monte Carlo samples which were drawn by resampling of observed values assuming a non-uniform distribution of values. The relative contribution of a factor both alone and in combination with other factors (*L* with season and *L* with sex) was calculated.

Besides prevalence, two other quantitative descriptors of the *S. solidus* population were used to evaluate the potential effect of different habitat choice: mean intensity (*MI*) and a combined parasite:host biomass ratio (parasite index, *PI*). Mean intensity was calculated separately for both sampled habitats as a mean number of tapeworms per infected host and intensity (*I*) per infected stickleback (parasites per host). *PI* was calculated as

1 $$PI=P/H$$

where *P* is the total wet weight of the parasites and *H* is the wet weight of the host with parasite (Pennycuick 1971).

To test if (a) *I* or (b) *PI* was influenced by habitat, season, sex, *L*, or the interaction of *L* with season and sex, the following GLM (Sachs 1997) was used:

2 $${Y}_{\mathrm{ijklmn}}=\mu +{\alpha }_{\mathrm{i}}+{\beta }_{\mathrm{j}}+{(\alpha \beta )}_{\mathrm{ij}}+{ \gamma }_{\mathrm{k}}+{\delta }_{\mathrm{l}}+{\varepsilon }_{\mathrm{m}}+{(\beta \varepsilon )}_{\mathrm{jm}}+{\zeta }_{\mathrm{ijklmn}}$$

where *Y*_ijklmn_ is *I* or *PI*; *µ* is the overall mean, *α*_i_ denotes season, *β*_j_ is *L*, (*αβ*)_ij_ is the interaction between season and *L*, *γ*_k_ represents year, *δ*_l_ is habitat (pelagic or benthic zone), *ε*_m_ is sex (male or female), (*βε*)_jm_ is the interaction between *L* and sex, and *ζ*_ijklmn_ is the random residual error. For *PI*, a square root transformation was made to meet the assumption of normal distributed residuals. A Box-Cox-transformation of the *I*-dataset was necessary due to residual deviation from model assumptions, leading to a lambda of − 2. Post hoc comparisons were made with Student’s *t* test if only two groups were compared or by building contrasts which were Bonferroni corrected.

All statistics were run on JMP Pro 15.1.0 (64 bit, SAS Institute).

### Availability of data and materials

The datasets used and analysed during the current study are available from the corresponding author on reasonable request.

## Results

### Size and abundance of sticklebacks

In total, of all analysed sticklebacks, 659 individuals (23.0%) were infected by *S. solidus*. All of those fish were individuals where gonads were developed (*L* > 51 mm) while no single tapeworm was found in a juvenile stickleback. Ninety percent of infected sticklebacks exhibited a length between 64 and 79 mm (median 72 mm), and the mean length of an infected stickleback was 71 mm (± 6 mm s.d.). 2.6% of infected fish (*n* = 17) had a mean length < 60 mm. During spawning time, the weight of an infected adult stickleback (*n* = 296) without the tapeworm was 3.3 g (± 0.8 g s.d.) while the weight of non-infected sticklebacks (*n* = 622) was significantly lower, at 3.1 g (± 0.8 g s.d.) (*t*-test, *P* < 0.001). Fifty-nine percent of infected sticklebacks caught during their spawning period were females, and 41% males. The corresponding figures for the non-infected sticklebacks in that period were 85% females and 15% males.

Two thousand sixty-nine of the 2871 analysed sticklebacks were caught with benthic gillnets, and 803 in the pelagic gillnets. The overall NPUE in the pelagic gillnets was low from January to September (Table 1). During this time, mainly adult individuals measuring between 60 and 75 mm were caught. Between May and July, the stickleback spawning season, only single sticklebacks were caught in the pelagic area, in some years not even a single fish was captured in this habitat during June. In Summer, between August and September, the first young-of-the-year individuals with *L* below 40 mm were observed, accompanied by some adults, as pelagic NPUE increased slowly. NPUE peaked in November and December and dropped thereafter (Table 1). Catches in the benthic gillnets showed a different trend, with the highest NPUE recorded during the stickleback spawning season between May and July (Table 1). During this time, exclusively adult individuals were caught. Between August and October, benthic NPUE dropped below 2.5 (Table 1), followed by a second sharp increase in late autumn, in November and December, and a second drop during winter times (Table 1). The first single individuals (*n* = 9) of young-of-the-year sticklebacks with *L* between 40 and 45 mm were recorded in the benthic gill nets during autumn (November).

**Table 1:** The mean catch per unit effort (NPUE in n/m^2^) of sticklebacks (± standard deviation s.d.) and minimum and maximum values (min–max) in Upper Lake Constance in the course of the year (2016–2019) in the pelagic and benthic zones

Season	Month	Mean NPUE pelagic (± s.d.)	Min–max pelagic	Mean NPUE benthic (± s.d.)	Min–max benthic
Spring	April	0.15 (± 0.18)	0–0.35	3.57 (± 4.30)	0–9.73
Spring	May	0.03 (± 0.02)	0–0.04	58.99 (± 30.70)	25.0–86.79
Summer	June	0.05 (± 0.06)	0.01–0.14	11.81 (± 17.77)	1.91–38.42
Summer	July	0.05 (± 0.03)	0.01–0.09	12.90 (± 20.13)	0.56–42.72
Summer	August	0.13 (± 0.10)	0.04–0.28	2.31 (± 3.33)	0.07–7.28
Summer	September	0.09 (± 0.05)	0.02–0.13	1.44 (± 2.45)	0.02–5.12
Autumn	October	0.24 (± 0.39)	0–0.7	0.10 (± 0.13)	0–0.26
Autumn	November	1.10 (± 1.27)	0.1–2.53	79.06 (± 14.19)	0–207.37
Autumn	December	7.65 (± 6.51)	0.89–13.89	5.98 (± 9.80)	0.17–17.31
Winter	January	0.25 (± 0.17)	0.06–0.39	0.60 (± 0.52)	0.13–1.18
Winter	March	0.05 (± 0.06)	0–0.14	1.02 (± 1.35)	0–2.87

### Size of tapeworm and prevalence

**Fig. 3:**
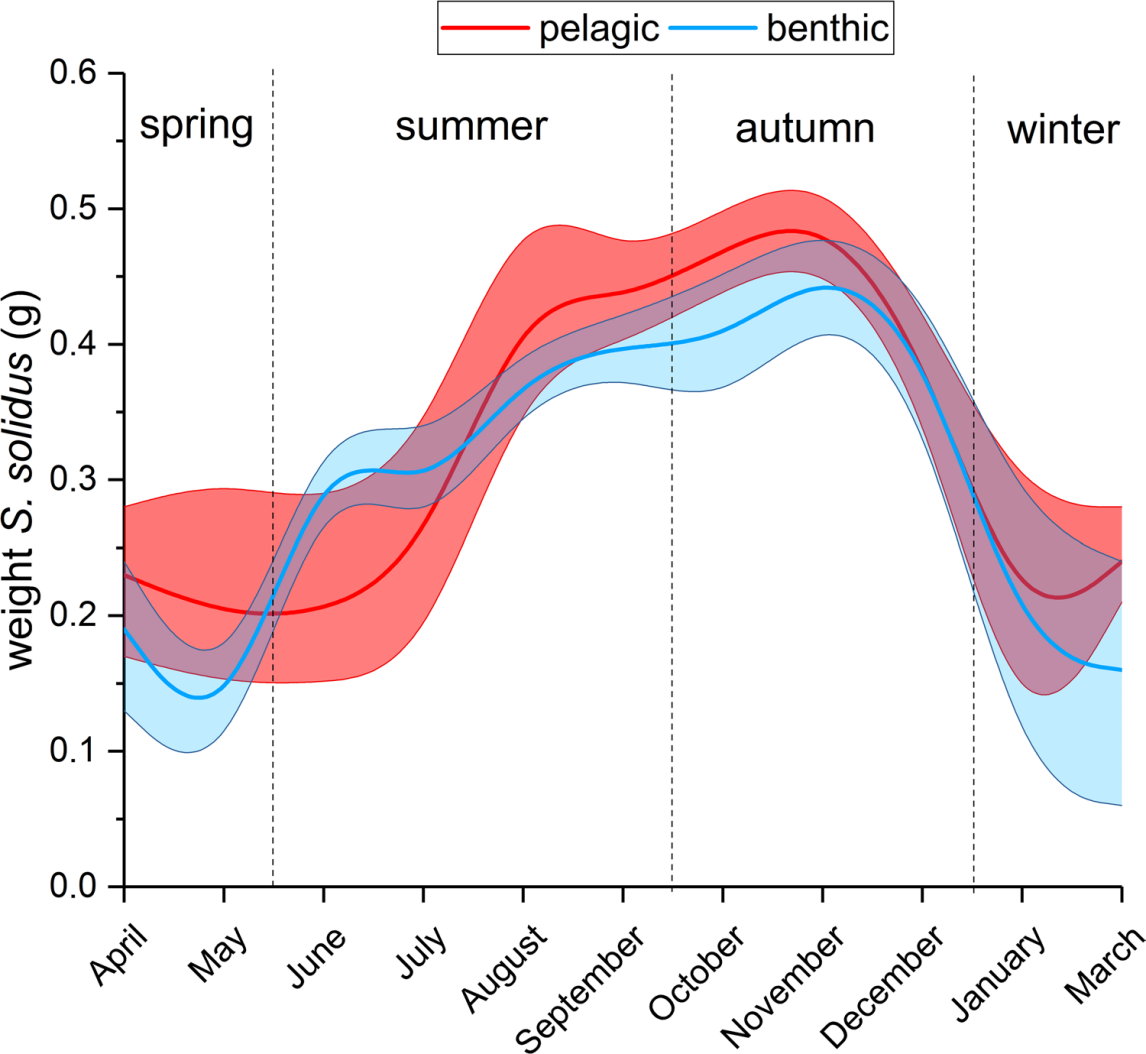
Spline-curve (polynomial interpolation) of mean weight of *Schistocephalus solidus* in the pelagic and benthic zones. Solid lines are the interpolated mean values and the shaded areas represent upper and lower 95% confidence intervals during the course of the year in Upper Lake Constance (2016–2019)

The size of *S. solidus* in both habitats was largely comparable: the smallest individuals, some with a weight below 0.1 g, occurred in spring (May) (Fig. 3). During the summer months, the weight of *S. solidus* increased and the largest individuals with a mean weight > 0.40 g were found in autumn (November) (Fig. 3). From December on and during winter, the mean weight of *S. solidus* decreased and in January and March only small individuals (mean weight around 0.2 g) were found (Fig. 3).

**Fig. 4:**
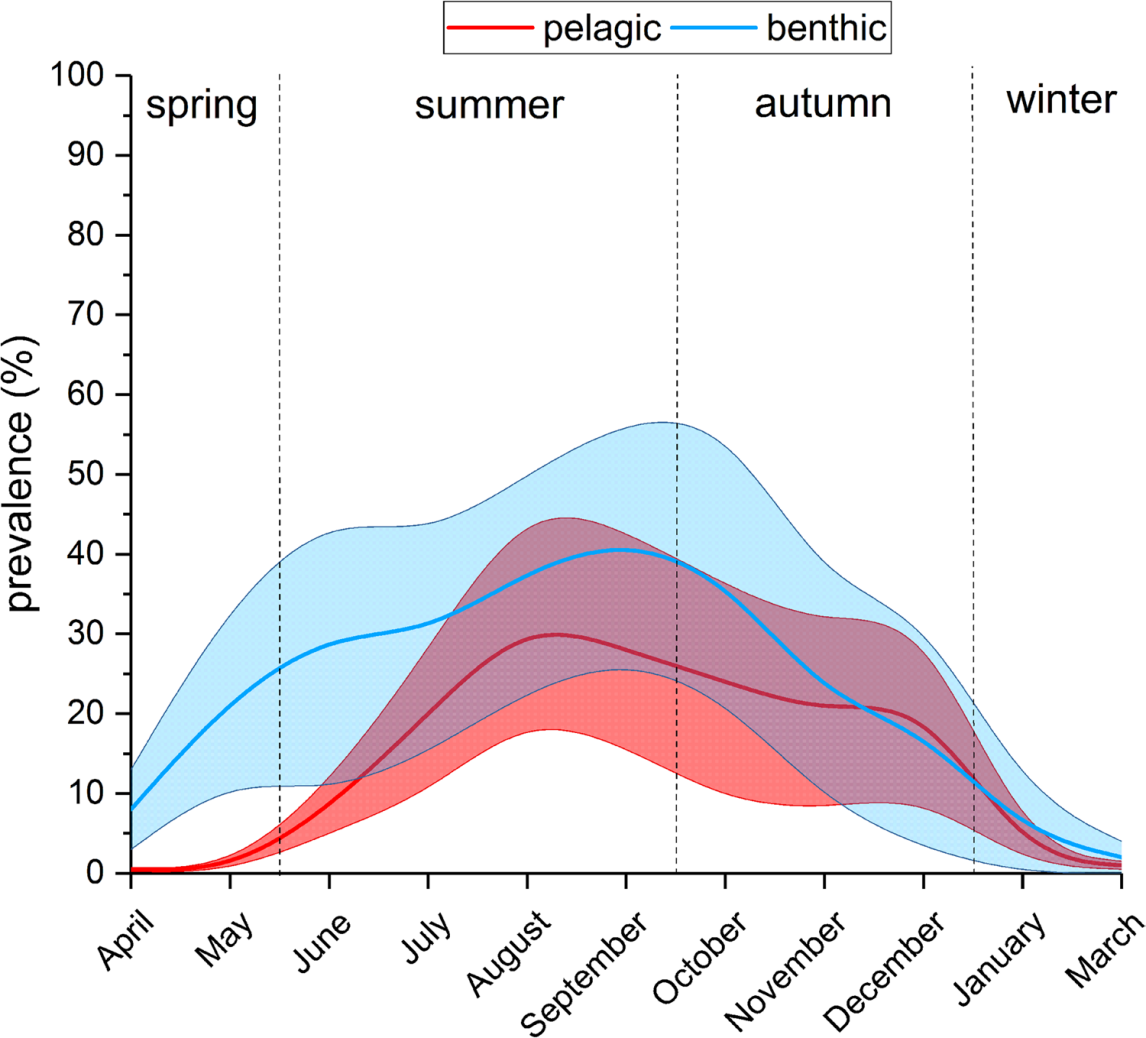
Spline-curve (polynomial interpolation) of mean prevalence with *Schistocephalus solidus* of sticklebacks in the pelagic and benthic zones. Solid lines are the interpolated mean values and the shaded areas represent upper and lower 95% confidence intervals during the course of the year in Upper Lake Constance (2016–2019)

Parasite prevalence in adult sticklebacks caught in the pelagic zone increased from nearly zero between January and May to a peak in late summer and kept relatively stable for the rest of the year (Fig. 4). In the benthic habitat, prevalence followed a different pattern. An initial peak occurred during the stickleback spawning season during spring, followed by a decrease during summer and second peak in autumn (Fig. 4).

**Fig. 5:**
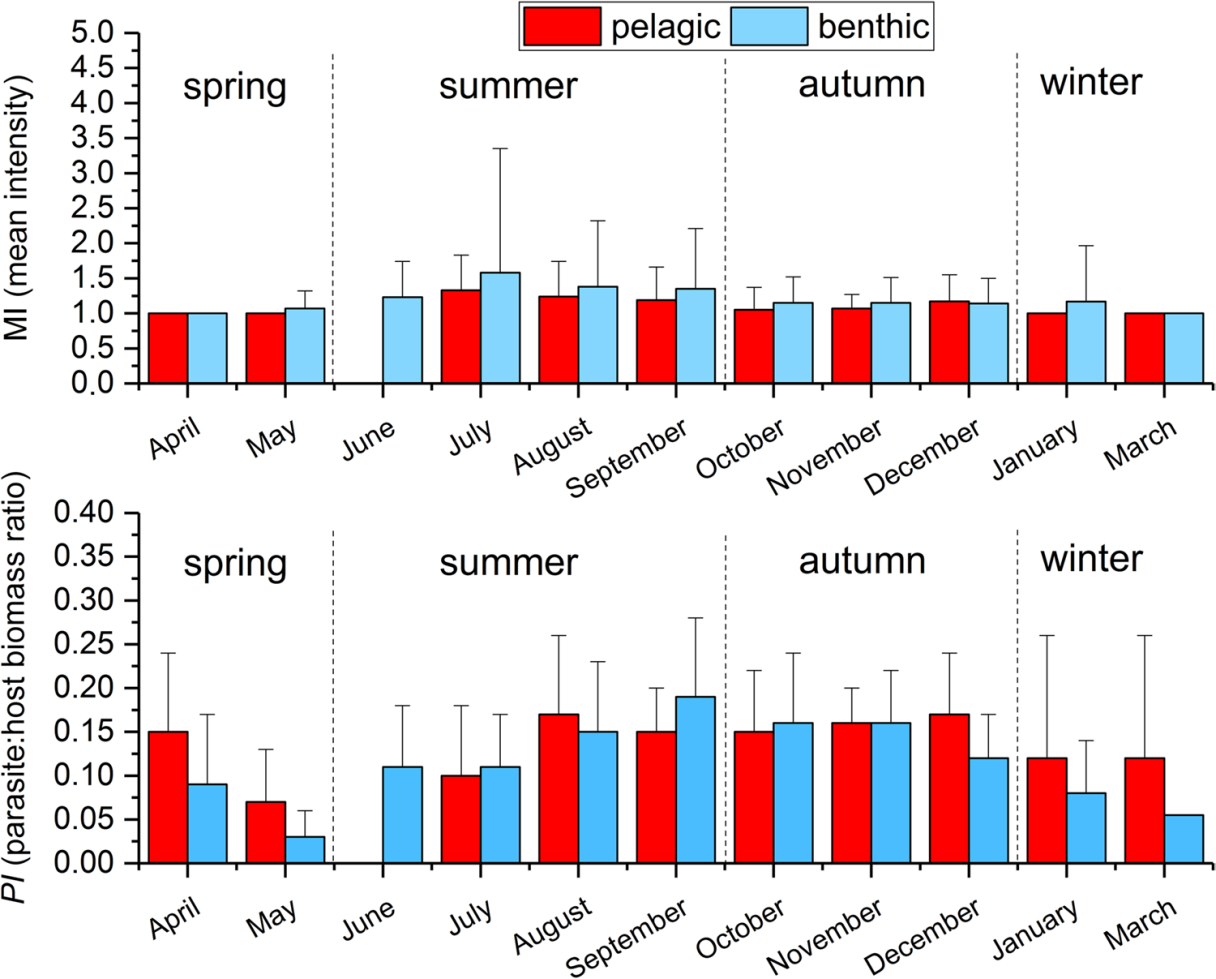
Prevalence with *Schistocephalus solidus* of female and male sticklebacks of different length classes in Upper Lake Constance (2016–2019)

The overall prevalence in the benthic zone (24.8%) was distinctly higher than in the pelagic zone (15.0%). However, the nominal logistic model (*n* = 2424, *P* < 0.0001) revealed that this difference was not linked to habitat (*P* > 0.05) but to stickleback size (*P* < 0.001). Furthermore, prevalence between sexes was significantly different (*P* < 0.001), seasons differ strongly (*P* < 0.001), and during the study course a linear reduction in prevalence became obvious (*P* < 0.001) (Table 2). Overall, the impact of *L* on prevalence had the highest effect strength (Table 2) and a positive correlation between *L* and prevalence was observed (as longer the stickleback as higher is the chance of infection). Prevalence in summer and autumn was significantly higher compared to winter and spring (*P* < 0.05). Sex showed a higher effect strength (compared to habitat) and males were significantly higher infected than females (Table 1). Males over 7 cm *L* showed prevalence > 50%; females of the same length classes showed prevalence values below 40% (Fig. 5). Furthermore, year had a significant impact on the outcome of the model (*P* < 0.001) and a negative correlation with ongoing sampling was observed (prevalence was constantly decreasing from 26.6% in 2016 to 7.2% in 2019). The interaction of season and sex with *L* showed minor *P*-values (Table 1) and had negligible impact on the outcome of the model.

**Table 2:** The significance, effect strength, and estimates and standard error (SE) of effect strength of different parameters on the prevalence of sticklebacks infected with *Schistocephalus solidus* in Upper Lake Constance (2016–2019)

Parameter	Significance/correlation^a^	Total effect strength*	SE total effect strength	Estimates
Total length	xxx/ +	0.361	0.01	0.87
Season	xxx/spring − , summer + , autum + , winter −	0.343	0.008	n.a
Sex	xxx/male + , female −	0.280	0.008	− 0.47
Year	xxx/ −	0.137	0.005	− 0.33
Habitat	n.s./benthic − , pelagial +	0.009	0.001	− 0.06
Total length × sex	x/male + , female −	n.a	n.a	n.a
Total length × season (summer)	xx/ −	n.a	n.a	n.a

^a^xxx = *P* < 0.0001; xx = *P* < 0.001; x = *P* < 0.05; + , positive correlation; − , negative correlation; *n.s.*, not significant; *n.a.*, not applicable; *dimensionless proxy assessing the relative impact of variables in the model formula by Monte Carlo simulations

### Intensity of infection

**Fig. 6:**
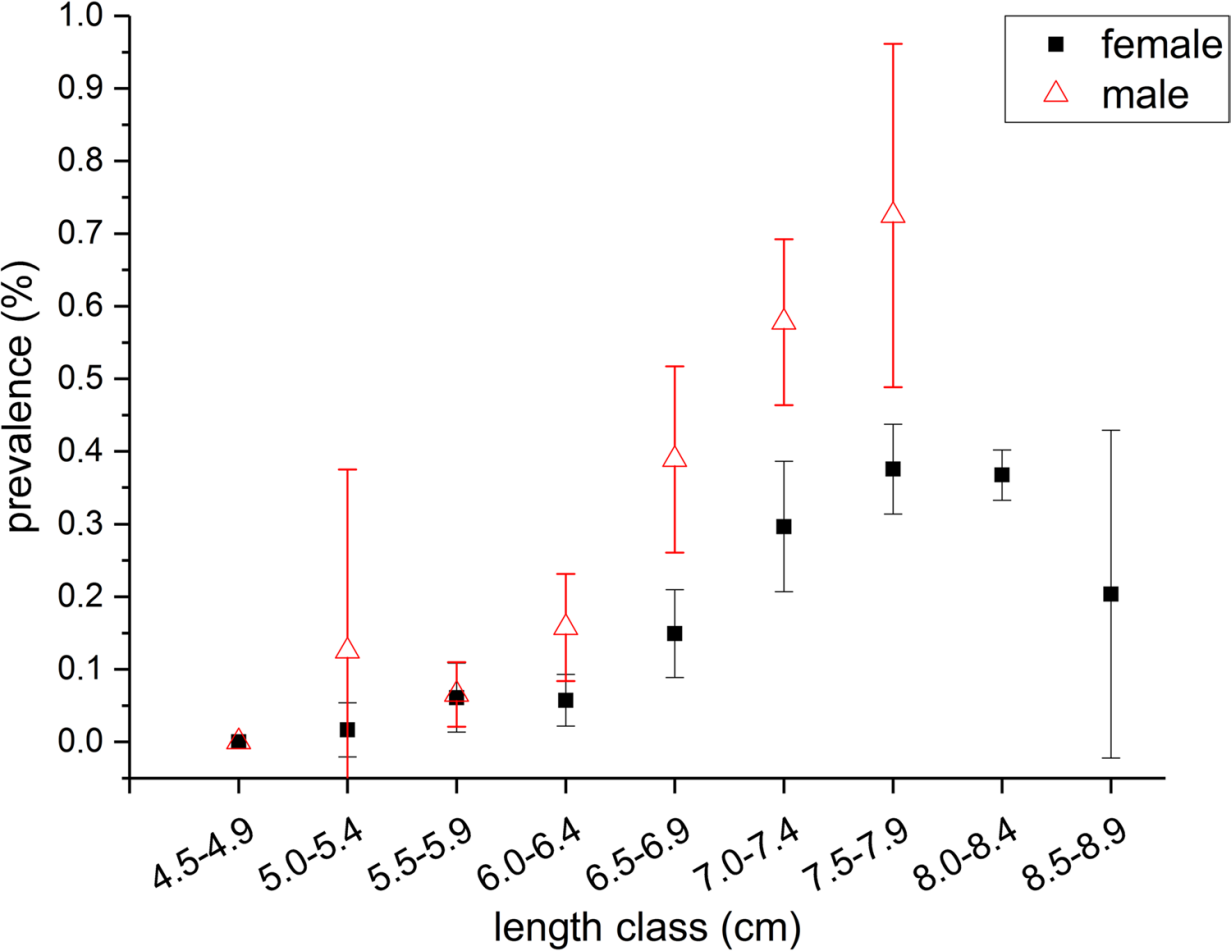
Mean intensity of infection (upper figure) and parasite:host biomass ratios (lower figure) (mean + s.d.) of *Schistocephalus solidus* infection in sticklebacks from Upper Lake Constance (2016–2019)

The mean intensity of *S. solidus* infection was comparable between habitats and essentially stable during seasons, with the mean value only exceeding 1 during single months in summer (Fig. 6). The model (*r*^2^_adjusted_ = 0.04, degrees of freedom (d.f.) = 354, *P* = 0.002) revealed that neither habitat, sex, *L*, year, nor the interaction of season and sex with *L* had an influence on intensity. Only season impacted *I*; here the difference between summer (increasing) and spring (decreasing) was significantly different (contrast test, *P* < 0.01).

Mean *PI* never exceeded 0.2 at any time in the study period (Fig. 6), independent on sampled habitat. The highest *PI* values for both habitats were recorded between August and December (Fig. 6). The GLM (*r*^2^_adjusted_ = 0.36, d.f. = 354, *P* < 0.001) revealed that season had the strongest effect on *PI* (Table 3). Post hoc comparison showed that all seasons had significantly different *PI*-values (contrast test, *P* < 0.05). Other significant parameters, but with low effect strength on *PI*, were habitat and *L* as well as the interaction of *L* with season and sex (Table 3). According to the GLM, the other parameters, year and sex, did not influence *PI* (Table 3).

**Table 3:** The significance, direction of correlation, scaled estimates, and standard error (SE) and *t*-values of scaled estimates of different parameters on the monthly *PI* of sticklebacks infected with *Schistocephalus solidus* in Upper Lake Constance

Parameter	Significance/correlation^a^	Scaled estimates^a^	SE of scaled estimates	*t*-values
Season — spring	xxx/ −	0.14	0.008	− 16.10
Season — summer	xxx/ +	0.03	0.006	4.38
Season — autumn	xxx/ +	0.08	0.007	10.23
Season — winter	xx/ +	0.04	0.010	3.65
*L*	x/ −	0.04	0.009	− 2.30
Habitat — pelagic	x/ +	0.01	0.005	2.54
Habitat — benthic	x/ −	0.01	0.005	− 2.54
Sex — male × *L*	x/ +	0.04	0.008	2.34
Sex — female × *L*	x/ −	0.04	0.008	− 2.34
Season — winter × *L*	xx/ +	0.08	0.013	3.19
Season — spring × *L*	n.s	n.a	0.013	− 1.20
Season — summer × *L*	n.s	n.a	0.011	− 0.32
Season — autumn × *L*	n.s	n.a	0.012	− 1.94
Year	n.s	n.a	0.005	− 1.45
Sex	n.s	n.a	0.005	1.11

^a^xxx = *P* < 0.0001; xx = *P* < 0.001; x = *P* < 0.05; + , positive correlation; − , negative correlation; *n.s.*, not significant; *n.a.*, not applicable

## Discussion

Contrasting environmental conditions can exert a significant local effect on species behaviour and evolution, and during this process, traits linked to species interactions including host-parasite relationships can evolve rapidly (Anaya-Rojas et al. 2016). Inducible tactics that pay off when the cost of infection and the probability of being infected are high might provoke adaptive change in the life history of a host (Forbes 1993). In ULC, as in other large lakes, environmental conditions differ strikingly between benthic and the pelagic habitats, resulting in different communities of algae, zooplankton, and fish (Rinke et al. 2009). In consequence, the hypothesis arose that a difference in parasite infection risk seems likely and could be a driver in ULC for the invasion of the sticklebacks into a formerly unoccupied habitat, the pelagic zone. However, the results presented here did not support this hypothesis, as other factors were much more influential on stickleback infection. Indeed, although a difference in prevalence between sticklebacks was observed between habitats, the effect was not influenced by locality but rather length and differences between sex, year, and season. Moreover, the infection risk in the pelagic habitat appears to be even slightly higher, and contradicts the initial hypothesis of this study that the outmigration into the pelagic zone could be triggered by infection avoidance. This is in line with the zooplankton distribution in ULC and the probability of sticklebacks to consume an infected first intermediate host, like *Cyclops* sp. (Wedekind and Milinski 1996), because cyclopoid copepods are one of the most common zooplankton taxa in the pelagic zone of ULC (Boit et al. 2012; Straile 2015). In other words, the difference of the infection risk between the pelagic and benthic habitat could not explain the recent unexpected migration activity of the sticklebacks in ULC. However, the data presented here points to another factor, which might stimulate or support stickleback migration: the spawning activity of whitefish in the pelagic area. In this study, sticklebacks are conspicuously present in the same areas as spawning pelagic whitefish, which was observed during November and December when the highest NPUE were recorded in the pelagic zone. The concurrent movement of the sticklebacks into the area where whitefish spawn, together with the information from recent studies which demonstrate that ULC sticklebacks are effective predators of whitefish larvae and can successfully forage on whitefish eggs (Roch et al. 2018; Ros et al. 2019; Baer et al. 2021), offers a biological trait to explain the invasion of the pelagic zone. Consequently, these high-energy food sources could be a main driver for the observed spreading of sticklebacks into the pelagic zone in ULC. However, future studies addressing this relationship in more detail are needed.

This situation observed in ULC is rare but not unique; therefore, answering this question could also aid understanding of stickleback invasion processes in other water bodies. A comparable situation has recently been described in the Baltic Sea (Bergström et al. 2015). In contrast to Lake Constance, the stickleback population is native there, but a population increase in the pelagic zone has led to comparable reduced stocks of other fish, which have been linked to stickleback predation on eggs and larvae of European perch *Perca fluviatilis* L. and pike *Esox lucius* L. (Ljunggren et al. 2010; Byström et al. 2015; Bergström et al. 2015). Compared to the situation in ULC, the abundance of sticklebacks in the Baltic is high and stable — even prompting interest from the commercial pelagic fishing fleet to start exploiting sticklebacks for fishmeal and biogas production (Bergström et al. 2015). Similar fisheries management options could also be appropriate for ULC as the stickleback population appears to be at a constantly high level (Eckmann and Engesser 2019; Gugele et al. 2020). This is somewhat surprising as sticklebacks are more generally known for periodic mass development (Nümann 1972) than persistent dominance of major habitats. One explanation for the high and stable abundance in ULC is the apparently minor impact of *Schistocephalus solidus* infection. This situation is most likely related to the life cycle of the host population in ULC. In studies with high impacts of *S. solidus* on sticklebacks density and no hyperabundant population levels (McPhail and Peacock 1983; Heins et al. 1999), most sticklebacks died in late autumn, having lived less than 1 year. In contrast, sticklebacks in ULC have a life expectancy of minimum 2 years. Larvae leave the nest during summer and migrate into the pelagic zone in large numbers (Gugele et al. 2020). Here they grow and juveniles up to 40 mm were found during September and October. Spawning sticklebacks in ULC exhibited a length of more than 60 mm, suggesting that they need to survive at least one winter in order to build nests, spawn, and perform parental care during their second summer. This is in line with several other studies where sticklebacks live longer than 1 year and spawn during their second summer as 1 + fish with *L* of > 55 mm (Craig and FitzGerald 1982; Dufresne et al. 1990; Moser et al. 2012). Moreover, in stickleback with *L* of < 55 mm, no *S. solidus* were found. This is in line with existing knowledge, because although sticklebacks can become infected already as young fish (Milinski and Christen 2005), most of them probably do not acquire infections until significant time after hatching (Heins et al. 2011). According to different studies (Heins et al. 1999, 2011), the earliest infections occur from mid-July through to late August, depending on the date of hatching. *S. solidus* allows its host to further grow after infection, in order to reach its definitive size (Milinski and Christen 2005), and large mortality of young-of-the-year sticklebacks in habitats where sticklebacks live longer than 1 year is rare (Heins et al. 2011). Nevertheless, based on the small sample size of analysed juvenile sticklebacks in ULC, the prevalence of this age class could be underestimated in the present study.

Shortly before the spawning season of sticklebacks, the prevalence was distinctly low and raised only marginally until the end of the spawning season and time of parental care. Thus, the major adverse effects of *S. solidus* in ULC were almost exclusively confined to post-reproductive adults. In accordance with McPhail and Peacock (1983), an effective evolutionary adaption for the plerocercoids is to only exert adverse effects on their host after they have reproduced. This suggests that a collapse of the stickleback population in ULC due to parasite-induced mortality is not likely. However, the question whether in former times the parasite was as abundant as today and had then induced a population crash before spawning could not be answered as prior stickleback invasion no specific monitoring of sticklebacks and their infection status were conducted. However, moribund sticklebacks in cold season have never been reported which might have probably been linked to such a case.

However, the presented data provide strong evidence for a majority parasite-induced mortality of *S. solidus*–infected sticklebacks in winter in ULC. The weights of plerocercoid larvae increased from spring to autumn, with an abrupt decrease following winter, when suddenly only small *S. solidus* individuals remained. As the parasite cannot be eliminated by the host, likelihood is that almost all hosts harbouring large parasites are dying rather abruptly at the end of autumn, accounting for the sudden decrease in weight seen. Mutually contributing factors are thought to be responsible for this observation. The first is a strong increase in pathogenicity of the tapeworm with its size (Ness and Foster 1999). Concurrently, living conditions become increasingly challenging in winter, for example with food scarcity (Pennycuick 1971). In combination, these lead to increased mortality rates during autumn and early winter (Threlfall 1968; Pennycuick 1971). At the same time, sticklebacks are probably more prone to predation and having been weakened by the parasite they have reduced swimming speed and manoeuvrability (Giles 1983; Jolles et al. 2020) becoming easier to catch (Ness and Foster 1999; Barber et al. 2004). In Walby Lake, Alaska, USA, a prevalence above 80% resulted in a parasite-induced population crash of sticklebacks (Heins et al. 2010). However, since the abundance of sticklebacks in ULC has now maintained a consistent extraordinary high level for eight consecutive years (Eckmann and Engesser 2019), the observed parasite-induced mortality does not seem to carry implications on population level. In other words: the highest yearly prevalence observed in this study of 26.6% (2016) will theoretically decrease the standing stock of sticklebacks in ULC (Gugele et al. 2020) by roughly about 31 million individuals, but more than 84 million (uninfected) sticklebacks will be not impacted by *S. solidus*. Therefore, even if millions of sticklebacks die due to the infection with *S. solidus*, the reproduction rate of sticklebacks will likely persist at a high level, as reproduction appears independent on mortality.

In the present study, infected fish were significantly heavier than non-infected fish during spawning time. This finding is in line with the outcomes of Heins et al. (2004), where nine-spined stickleback *Pungitius pungitius* (L. 1758) females parasitised by *Schistocephalus pungitii* (Dubinina 1959) exhibited better body condition compared to uninfected fish. In another study, the slope of the ovarian mass:body mass relationship was significantly higher in experimentally infected fish than in uninfected fish, and infected fish had greater ovarian masses than uninfected fish of the same size (Barber and Svensson 2003). Nevertheless, how infection with *S. solidus* impacts the reproduction success of sticklebacks in ULC is currently unknown. The larger size of infected fish suggests that energy allocation, and therefore ovarian mass, was minimally affected by the parasite. However, the high food consumption responsible for the larger size had increased risk of infection due to higher consumption of parasite vectors, i.e. copepods. Here, it should be taken into account that infection by *S. solidus* could be associated with reduced reproductive fitness (Ritter et al. 2017). The main reproductive processes identified that are impacted in parasitised female sticklebacks include potentially slower progress through the clutch production cycle (Heins et al. 1999) and a delay in ovarian maturation which may lead to a de-synchronisation of spawning (Meakins 1974). This could be the case in ULC too; however, here possible impacts on reproduction will be low as noticeably more than 50% of the ULC population recorded during the spawning season were uninfected, and additionally no effect on stickleback recruitment success occurred (Eckmann and Engesser 2019). Further work is required to determine the consequences of varying size among infected and non-infected species in Lake Constance.

In line with other studies, prevalence in ULC was dependent on sex (Tierney et al. 1996; Reimchen and Nosil 2001). However, in ULC, males showed a significantly higher prevalence than females whereas other studies reported the opposite (Tierney et al. 1996; Reimchen and Nosil 2001). In these studies, females consume more copepods, the primary host of *S. solidus* second larvae stage. This provides strong evidence for differential exposure to infected prey between the genders. Nevertheless, that females show higher prevalence is not always the case. The opposite has been shown in a lake in Canada, where during a 15-year study males consumed more copepods than females in certain years, without affecting the prevalence of the whole population (Reimchen and Nosil 2001). This suggests that temporal shifts in relative parasitism are possible and are most likely caused by shifts in the diet resulting in changes in exposure to infected prey items. It is assumed that in the present study or during the studied years males fed more on copepods than females; therefore, *S. solidus* infections in ULC tended to be male-biased. It has to be taken into account that males are responsible for building the nest and guarding eggs and fry; therefore, the time they spend in the littoral zone is likely longer than females. Thus, one main food source of sticklebacks like pelagic living daphnia (Bretzel et al. 2021) is available for males only for a shorter time frame compared to females, and alternative diets, like copepods, are chosen, leading to a higher infection risk. Nevertheless, the reasons for this sex-dependent feeding and therefore infection pattern must be explored in future scientific studies.

From the present data, the study did not support the hypothesis that differences in parasite burdens have the potential to contribute to divergent selection on life history and generate genetic divergent sub-populations in Lake Constance (MacColl 2009). The opposite was seen here, where similar prevalence in the pelagic and benthic zones suggests a highly comparable life cycle and feeding ecology of sticklebacks occupying the two habitats. Future genetic studies could reveal if the sticklebacks of Lake Constance are one large and very mobile population, as the present study suggests, or if different genetic sub-populations exist as hypothesised elsewhere (Moser et al. 2012; Hudson et al. 2021). These studies should be accompanied with morphological measurements, because in several lakes in coastal British Columbia sticklebacks have undergone parallel diversification, resulting in the formation of two specialised and morphological different species, in a benthic and limnetic one (Harmon et al. 2009). If such a parallel diversification happened in Lake Constance, too, and the adaptation to two different habitats took part in the last decades, different phenotypes should be detectable, as phenotypic plasticity in sticklebacks is tightly connected to different ecological niches (Svanbäck and Schluter 2012).

Other studies hypothesised that a reduction of piscivorous fish species in the open waters of a lake, like a reduced stock of arctic charr, could be the explanation for the observation of migrating sticklebacks into this “predator free” zone (Dahl-Hansen 1995). However, the invasions of sticklebacks in the pelagic zone of ULC could most likely not have been explained by predator avoidance behaviour: On the one hand, no hint exists that the density of piscivorous fish is decreasing in recent years (the commercial yield of predatory fish in the open water like arctic charr *Salvelinus umbla*, pike *Esox lucius*, and migratory brown trout *Salmo trutta* is relatively constant in ULC, see www.IBKF.org); on the other hand, the density of fish eating birds in the pelagic zone (great crested grebe, *Podiceps cristatus*) is even increasing in recent years (own observation).

In conclusion, the invasion of sticklebacks in the pelagic zone of ULC is unlikely to be driven by an uneven risk of infection by *S. solidus* or predation between the littoral and pelagic zones. The possibility to forage during the spawning time of whitefish on the high-energy food source “whitefish eggs”, and later their larvae, seems to be a more likely driver of the invasion (Baer et al. 2021). Furthermore, major adverse effects of *S. solidus* in ULC were confined mainly to post-reproductive adult sticklebacks. Therefore, the impact of the parasite on the population dynamics of its host is likely compensated by the sticklebacks’ high fecundity and recruitment potential. Unlike in other waters where parasite impact has restored normality of abundant densities, the impact of *S. solidus* infection in ULC is unlikely to reduce the hyper-abundant stickleback population.
